# Clinic of Professor Mütter

**Published:** 1844-02-10

**Authors:** H. T. Child


### CLINIC OF PROFESSOR MÜTTER.

*December* 13, 1843.

(Reported by H. T. Child.)

### CASE OF LUPUS.

The patient now before you has suffered for six years from an ulcer which involves the inner canthus of the eye and a portion of the nose. The ulcer commenced as a small tubercle, which opened, and the ulcerated surface has gradually increased, until at present it equals in size a quarter of a dollar. Its margins are elevated, hard, everted and whitish—the granulations forming the surface are firm, irregular in shape, and scarcely sensible to any impression—the secretion is glairy and alkaline, without much fetor, and there is often a sharp lancinating pain from which the patient suffers considerably. The adjacent soft parts for two or three lines are reddish, hard and swollen, but the lymphatics are not diseased, nor has the general health of the patient suffered materially. He is 45 years old, and has no recollection of any such disease having existed in any member of his family—nor can he trace it to any manifest cause in his own case. We shall give him five drops of Donovan's Solution three times a day in a wineglassful of water, and apply "Adams' paste" to the ulcer.

*Remarks.*—This is obviously a case of Lupus, a disease evidently recognised by the ancients, although the views they entertained relative to its pathology and treatment were crude and incorrect. It received the appellation of Lupus from Willan and Bateman, but various other terms have recently been introduced, and in consequence of this the student often finds difficulty in deciding which to select. I wish you to bear in mind, however, that the herpes excedens, dartre rongeante, leperoid tumour, and lupus mean one and the same thing.

The seat of this disease is usually some part of the face, and especially the nose, but it may be developed in other regions of the body. Usually it commences in the skin, but the mucous membranes of the nose or cheek may be first attacked.

According to Biett, Lupus presents in its origin three distinct forms. In the *first*, the ulcer extends along the surface, and rarely, if ever, involves the deeper seated tissues. In the *second* the reverse of this obtains, and it penetrates rapidly, destroying all the tissues in its vicinity. In the *third*, there is decided and extensive *hypertrophy* of the parts involved.

In each variety the symptoms are peculiar. When, for instance, Lupus is confined to the superficial layer of the skin, we have at first merely redness of the surface, in spots of different sizes—from which the cuticle separates in the shape of thin scales—leaving the surface smooth, shining, red, and resembling the cicatrix of superficial burns. There is no pain—nor have we any tubercles or scabs. In other cases the disease commences by the development of small, soft reddish tubercles, which in time coalesce, increase in size, and ulcerate, the ulcer being covered with a brownish crust like the bark of a tree, and whenever this crust is removed the ulcer extends, and larger scabs and crusts form. In this variety the destruction of the surface is often very extensive.

In that form of Lupus which is characterized by a deep ulcer, the disease commences with redness and tumefaction—the tumefied part soon becomes violet coloured or livid, and then ulcerates.

The ulcer is covered with a thick scab, which falls off, to be succeeded in a few days by one of larger size. The nose is usually the seat of this form, and is often destroyed in part or entirely.

When Lupus is complicated with hypertrophy the disease begins by the development of several soft, indolent tubercles, which run together at their bases, and do not ulcerate. They gradually increase, until the part attacked assumes in many casen an enormous size—from the surface of which the cuticle is constantly peeling off, without the formation of a distinct crust, scab or ulcer.

Lupus may continue a nmmber of years without giving the patient much trouble, but often it runs its course in a few months. It is for the most part a disease of youth, and must not be confounded with the noli me tangere of old persons.

With respect to the character of its causes, it must be confessed that we know very little. It is often found in scrofulous individuals, and those whose cutaneous surfaces are liable to eruptive diseases, particularly erysipelas.

Lupus has been mistaken for acne rosacea, tubercular lepra, elephantiasis, noli me tangere, and syphilitic ulcerations, but a careful examination of the case will enable us readily to distinguish it from these diseases.

The prognosis is always unfavorable, inasmuch as it generally proves obstinate, and causes great deformity before our remedies can prevail against it. It cannot, however, so far as the life of the patient is concerned, be considered a dangerous disease.

In the treatment of lupus, general remedies rarely prove of much value, the affection being often purely local in its character. Still where any constitutional symptoms are present, it is essential to resort to such measures as shall cause their removal. I have sometimes derived much benefit from the iodide of potassium, when a scrofulous diathesis was manifest, and sometimes Donovan's Solution,—the liquor hydriodatis arseneci et hydrargyri,—will prove useful when the ulcer resists the usual local treatment.

The local treatment depends somewhat upon the nature of the case, but I caution you against resorting here to the application of the different—so called—*resolvent* washes and ointments. When lupus really exists, we may resort to one of three modes of operation:—First, cut out the disease entirely, and heal the flaps by the first intention. Second, cut out the disease, and supply its place with a flap of healthy skin taken from its vicinity. Third, apply some powerful caustic, which destroys the diseased part in a short time, causes a slough, and thus gives us a healthy surface on which granulations form. The caustics I prefer, are the chloride of zinc, or Adams' Paste, thus prepared

| | | |
|---|---|---|
| ℞. Arsenici Oxydi, | ʒj. | |
| Unguent. Cetacei, | ℥ss. | |
| Emplast. Cantharidis, | ʒiij. | |
| M. Ft. Ung. | | |

Or Adams' Powder, thus,

| | | |
|---|---|---|
| ℞. Arsenici Oxydi, | ʒj. | |
| Calcis, | ʒss. | |
| Cupri Sulphat. | ʒss. | M. |

A small quantity to be dusted upon the ulcer; after the slough is detached, simple dressings are sufficient. *(To be continued.)*

